# Preliminary evidence of increased striatal dopamine in a nonhuman primate model of maternal immune activation

**DOI:** 10.1038/s41398-019-0449-y

**Published:** 2019-04-12

**Authors:** Melissa D. Bauman, Tyler A. Lesh, Douglas J. Rowland, Cynthia M. Schumann, Jason Smucny, David L. Kukis, Simon R. Cherry, A. Kimberley McAllister, Cameron S. Carter

**Affiliations:** 1 Department of Psychiatry and Behavioral Sciences, University of California, Davis, CA USA; 2 California National Primate Research Center, University of California, Davis, CA USA; 3 The MIND Institute, University of California, Davis, CA USA; 4 Center for Genomic and Molecular Imaging, University of California, Davis, CA USA; 5 Department of Biomedical Engineering, University of California, Davis, CA USA; 6 Center for Neuroscience, University of California, Davis, CA USA

## Abstract

Women exposed to a variety of viral and bacterial infections during pregnancy have an increased risk of giving birth to a child with autism, schizophrenia or other neurodevelopmental disorders. Preclinical maternal immune activation (MIA) models are powerful translational tools to investigate mechanisms underlying epidemiological links between infection during pregnancy and offspring neurodevelopmental disorders. Our previous studies documenting the emergence of aberrant behavior in rhesus monkey offspring born to MIA-treated dams extends the rodent MIA model into a species more closely related to humans. Here we present novel neuroimaging data from these animals to further explore the translational potential of the nonhuman primate MIA model. Nine male MIA-treated offspring and 4 controls from our original cohort underwent in vivo positron emission tomography (PET) scanning at approximately 3.5-years of age using [^18^F] fluoro-l-m-tyrosine (FMT) to measure presynaptic dopamine levels in the striatum, which are consistently elevated in individuals with schizophrenia. Analysis of [^18^F]FMT signal in the striatum of these nonhuman primates showed that MIA animals had significantly higher [^18^F]FMT index of influx compared to control animals. In spite of the modest sample size, this group difference reflects a large effect size (Cohen’s *d* = 0.998). Nonhuman primates born to MIA-treated dams exhibited increased striatal dopamine in late adolescence—a hallmark molecular biomarker of schizophrenia. These results validate the MIA model in a species more closely related to humans and open up new avenues for understanding the neurodevelopmental biology of schizophrenia and other neurodevelopmental disorders associated with prenatal immune challenge.

## Introduction

Mounting evidence from human epidemiological studies suggests that exposure to a variety of viral or bacterial infections during pregnancy increases the risk for neuropsychiatric and neurodevelopmental disorders in exposed offspring^1^. The importance of the intrauterine environment is further demonstrated by a series of recent studies linking maternal inflammation during pregnancy, as indexed by plasma interleukin 6 (IL-6) concentrations, with offspring brain and behavior phenotypes relevant to neurodevelopmental and neuropsychiatric disease^2–5^. Animal model systems have established mechanistic links between prenatal exposure to infection, the subsequent maternal immune response and alterations in offspring neurodevelopment^6^. One of the most widely used agents in preclinical maternal immune activation (MIA) model research is polyinosinic-polycytidylic acid (PolyIC), a synthetic analog of double-stranded RNA, which triggers a potent immune response^7,8^. Rodent offspring born to PolyIC-injected dams exhibit changes in brain and behavioral phenotypes that bear resemblance to human disorders, including both autism spectrum disorder (ASD) and schizophrenia (SZ)^9,10^. The emerging consensus is that activation of the maternal immune system during pregnancy serves as a “disease primer” that, in combination with other genetic and/or environmental risk factors, may increase the risk for specific neurodevelopmental disorders^11^.

Although mouse models have laid the foundation for evaluating the effects of prenatal immune challenge on neurodevelopment, nonhuman primate models uniquely facilitate exploration of complex social and cognitive symptoms found in many human diseases^12,13^. The nonhuman primate model closely resembles humans in both behavior and neuroanatomical complexity and provides an opportunity to bridge the gap between rodent model systems and human patient populations^14^. Indeed, Coe and colleagues have demonstrated that nonhuman primate offspring prenatally exposed to viral or bacterial infections late in pregnancy exhibit alterations in brain and behavioral development^15,16^. To further explore the effects of the maternal immune response in the absence of a specific pathogen, we developed the first PolyIC-based nonhuman primate MIA model. Pregnant rhesus monkeys (*Macaca mulatta*) injected with a modified form of PolyIC over three days at the end of either the first or second trimester exhibited a transient but potent immune response and produced offspring that developed abnormal repetitive behaviors, reduced affiliative vocalizations, and altered immune regulation^17,18^. As they matured, the first trimester-exposed monkey offspring also exhibited inappropriate interactions with novel conspecifics and failed to attend to salient social cues in a highly translational eye-tracking paradigm^19^. The presence of abnormal repetitive behaviors paired with alterations in social development exhibited by MIA-treated offspring resemble features of several neurodevelopmental disorders, including both ASD and SZ.

Preclinical MIA models provide a unique opportunity to explore the neurobiology of aberrant offspring behaviors associated with maternal infection that may (or may not) cross current human diagnostic categories^20^. Mice and rats born to MIA-treated dams consistently show evidence of aberrant dopamine system development, including evidence of dopaminergic hyperfunction in the striatum^21–25^. Given that increased striatal dopamine is also a hallmark biological marker of SZ^26^, we initiated an in vivo positron emission tomography (PET) study in MIA-treated nonhuman primates to bridge the gap between rodent MIA models and clinical populations. MIA-treated and control animals were scanned at 3.5-years of age using [^18^F] fluoro-l-m-tyrosine (FMT) to measure presynaptic dopamine levels in the striatum. Although sex differences are emerging as an important factor in MIA model studies^27^ we had too few female offspring in the original cohort and therefore limited this initial PET pilot study to the 9 MIA-treated and 4 control males. Here we present preliminary evidence indicating that male nonhuman primate offspring born to MIA-treated dams exhibited increased striatal dopamine in late adolescence, thus extending findings from rodent MIA models into a species more closely related to humans.

## Methods

All experimental procedures were developed in collaboration with the veterinary, animal husbandry, and environmental enrichment staff at the California National Primate Research Center (CNPRC) and approved by the University of California, Davis Institutional Animal Care and Use Committee. All attempts were made (in terms of social housing, enriched diet, use of positive reinforcement strategies, and minimizing the duration of daily training/testing sessions) to promote the psychological well-being of the animals that participated in this research. Detailed methods from this cohort are available in our previous publications^17,19^ and have been updated below to reflect newly developed MIA model reporting guidelines^8^.

### Subjects and maternal immune activation (MIA) protocol

The MIA offspring (Table 1) were born to dams injected with 0.25 mg/kg synthetic double-stranded RNA (polyinosinic:polycytidylic acid [poly IC] stabilized with poly-l-lysine [poly ICLC]; Oncovir, Inc., Washington, DC) via intravenous (i.v.) injection while temporarily restrained by trained technicians at the end of the first trimester on gestational days (GD) 43, 44, 46 or at the end of the second trimester on GD 100, 101, 103. CON offspring were born to dams injected with saline at these same time points, or had no manipulation at all during pregnancy. Dams injected with poly ICLC exhibited a transient increase in IL-6 levels, temperature and sickness behaviors as previously reported^17^. PET imaging studies included all male control (CON; *n* = 4) and first trimester MIA exposed (*n* = 5) and second trimester MIA exposed (*n* = 4) offs.

**Table 1:** Experimental groups

Experimental group	Original MIA cohort (males, females)	PET imaging (males only)
1^st^ Trimester MIA (MIA^1^)	*n* = 6 (5m, 1f)	5
2^nd^ Trimester MIA (MIA^2^)	*n* = 7 (4m, 3f)	4
Saline Controls	*n* = 8 1^st^ Trimester (1m, 3f) 2^nd^ Trimester (2m, 2f)	3
Untreated Controls	*n* = 3 (1m, 2f)	1

### Rearing conditions and husbandry

All infants were born at term, raised with their mothers and provided three hours daily access to a social group consisting of four mother-infant pairs and one adult male to facilitate species-typical social development. Infants were raised in individual cages with their mothers where they had visual access to other animals at all times. Infants were weaned at 6-months of age, but continued daily peer group interactions through approximately 2-years of age. At the time of the current study, all animals were housed indoors in social MIA/CON pairs 24 h per day, 7 days per week. These pairs occupied two adjacent, age-appropriate laboratory cages where they had visual access to other animals. Animal rooms were maintained at 17.78–28.89 °C and on a 12/12 light/dark cycle (lights on at 0600). Subjects were fed twice daily (Lab Diet #5047, PMI Nutrition International INC, Brentwood, MO), provided with fresh produce biweekly, had access to water ad libitum and a variety of enrichment devices.

### Structural imaging

Structural MR imaging was performed prior to PET imaging at the University of California Davis Imaging Research Center using a 1.5 T GE Signa Horizon LX NV/I MRI system (GE Medical Systems, Waukesha, WI, USA). The protocol includes a T1-weighted SPGR (TR 27 ms, TE 6 ms, Flip Angle 30, matrix 256×256, FOV 160 mm, slice thickness 0.7 mm).

### Positron emission tomography (PET) imaging

Between 43.1 and 49.8 months of age, animals underwent PET scans using 6-[^18^F]fluoro-l-m-tyrosine ([^18^F]FMT). [^18^F]FMT was synthesized according to the method of VanBrocklin et al.^28^ as follows. Cyclotron-produced [^18^F]F2 (Siemens RDS111) was bubbled through a solution of N,O-Di-Boc-2-TMSn-m-tyrosine ethyl ester (ABX; 60 mg) in Freon (15 mL) in a vented pyrex reaction vessel at room temperature. The reaction mixture was passed through 2070 mg of silica gel in three cartridges (Waters Sep Pak Plus). The protected intermediate product was eluted from the cartridges in 25% diethyl ether/hexane solution. Pooled high activity fractions (10 mL) were added to 48% HBr (2 mL) and heated to 130 ^o^C. Ether/hexane was evaporated under a gentle stream of nitrogen, after which hydrolysis in HBr was continued for 5 min. After cooling, the crude product solution was partially neutralized in a solution of 50% w/w NaOH (0.45 mL) and HPLC solvent (0.55 mL). [^18^F]FMT was purified by HPLC, using a C18 column (Phenomenex Luna, 10 × 250 mm) eluted in 0.01% ascorbic acid/0.1% acetic acid (aq) at 5 mL/min (tR 16–17 min). HPLC-purified [^18^F]FMT was formulated with 10% disodium hydrogen phosphate (aq), 9% sodium chloride, and water to prepare a neutral solution of desired volume in 0.9% sodium chloride, then transferred with 0.22 µm filtration to a dose vial. All preparations of [^18^F]FMT (*n* = 16) demonstrated >99% radiochemical purity and >97% enantiomeric purity, by analytical HPLC (Waters Breeze; 220 nm UV detection; inline Bioscan RA detection). For radiochemical purity, [^18^F]FMT of measured activity was injected onto a C18 column (Phenomenex Luna, 4.6 × 250 mm) and eluted in 5% ethanol (aq) at 0.4 mL/min. Radiochemical purity was the fraction of total activity associated with the [^18^F]FMT peak (tR 8.8 min). For enantiomeric purity, [^18^F]FMT was co-injected with authentic L-FMT and D-FMT standards (ABX) onto a chiral column (Phenomenex Chirex D-penicillamine, 4.6 × 150 mm) and eluted in 2 mM copper (II) sulfate in 15% methanol (aq) at 1 mL/min (L-FMT tR = 22 min; D-FMT tRD = 29 min). Animal weights were measured on the day prior to the scan using the jump box method. On the day of imaging, the animal was sedated using 10 mg/kg ketamine (IM), followed by placement of two indwelling IV catheters into the cephalic and saphenous veins. The cephalic vein is used for the carbidopa and radiotracer injection while the saphenous vein is used for fluid therapy. [^18^F]FMT behaves similarly to [^18^F]FDOPA and is metabolized in the periphery, causing lower bio-availability in the brain^29^. To avoid this metabolism, carbidopa was administered as a peripheral decarboxylase inhibitor^29–33^. For these studies, carbidopa (5 mg/kg) was injected approximately 45 min prior to injection of [^18^F]FMT. Heart rate, respiratory rate, and SPO2 were monitored throughout the scan. Anesthetized animals were placed on a custom-built bed for Rhesus Macaque and placed headfirst into the microPET-P4 small animal PET scanner (Siemens Preclinical Solutions, Knoxville TN). This scanner has a reconstructed spatial resolution of ~2.0 mm^34^. First, a single pass transmission scan was acquired from each animal as a reference for attenuation and scatter corrections during image reconstruction. [^18^F]FMT was administered at approximately 0.5 mCi/kg. Once the radiotracer was injected, the IV catheter was removed and any residual activity in the syringe was measured. A 1.5 h dynamic emission scan was started approximately 5 s prior to injection of the ligand. The dynamic PET data consists of 24 frames of the following durations: 10 frames at 1 min each, 5 frames at 2 min each, 4 frames at 5 min each, and 5 frames at 10 min each. Images were reconstructed using the 3DRP reconstruction algorithm with a Hann filter (cut-off at 0.3 Nyquist) with attenuation and scatter correction applied. This protocol has been extensively validated for quantitative studies of the non-human primate brain.

### Image analysis

Dynamic data was analyzed using PMOD (PMOD Technologies Ltd., Zurich, Switzerland). A rough manual alignment of the PET images was performed before automated registration. Dynamic PET [^18^F]FMT images were registered to anatomical MRI images from each animal. All PET frames were summed and a Gaussian filter applied to smooth the data. The registration matrix was saved and subsequently applied to the dynamic, unsmoothed PET data. The Patlak reference tissue model was used to determine an index of influx (Ki) of the bilateral striatum for [^18^F]FMT studies using the cerebellum as the reference tissue^35,36^. For the Patlak analysis, a time cutoff value of 25 min was used for model fitting to provide the best sampling of dynamic data for linear fitting of the model. All brain regions of interest were defined on the co-registered MRI dataset by analysts blind to the treatment condition of the animals. For [^18^F]FMT Ki determination, bilateral caudate and putamen as defined by refs. ^37,38^ were manually traced utilizing the segmentation software ITK-snap^39^. No partial volume corrections were performed as the resolution of the striatum on the structural image in the NHP exceeded the resolution of the PET scanner. However, we determined that the tail of the caudate had insufficient resolution due to size and was therefore anatomically delineated from the head at its caudal extent and excluded from the manually defined ROIs^40^. Statistical analyses were conducted using IBM SPSS Statistics 25. Group differences in sample characteristics, including age and weight (Table 2), were tested with independent samples *t*-tests. In order to account for variability in the Patlak reference tissue model fit, group differences in FMT Ki were assessed using weighted least squares regression, with the inverse of the sum of squared residuals as weights. An estimate of the effect size was calculated using Cohen’s *d*. Data approximated a normal distribution according to Shapiro–Wilk tests (all *p* > 0.15). Additionally, data met criteria for equal variances according to Levene’s Test (weight: *p* = 0.43, age: *p* = 0.10, FMT Ki: *p* = 0.87).

**Table 2:** Age, weight, and [^18^F] FMT for PET sample

Experimental group	Age at scan in years (SD)	Weight at scan in kilograms (SD)	[^18^F]FMT index of influx (SD)
MIA	3.95 (0.10)	7.50 (0.99)	0.0204 (0.0020)
Controls	3.75 (0.16)	6.82 (1.30)	0.0183 (0.0021)

## Results

**Fig. 1:**
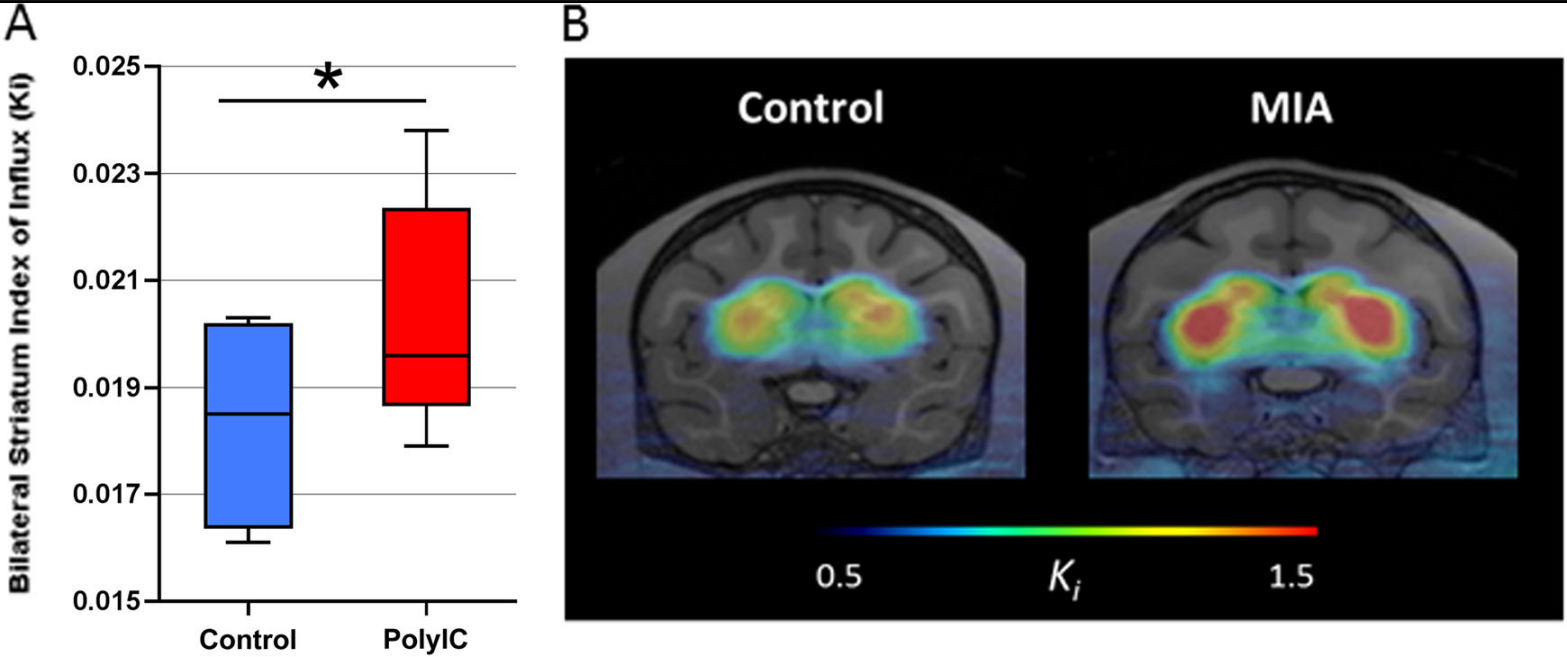
[^18^F]FMT index of influx in MIA-treated compared to control offspring. **a** Weighted least squares regression revealed that significantly higher [^18^F]FMT index of influx in MIA compared to control animals (F_1,11_ = 10.98, *p* = 0.007). **b** PET SUV images showing the striatal uptake of [^18^F]FMT. [^18^F]FMT PET images overlaid on anatomical MR images and are scaled to the same lower (0.5) and upper thresholds (1.5) as depicted in the color bar

First trimester and second trimester MIA exposed males were not significantly different in age (t(7) = 0.0238, *p* = 0.818), weight (t(7) = 0.113, *p* = 0.914), or [^18^F]FMT index of influx (F_1,7_ = 1.174, *p* = 0.314). Consequently, for all analyses, all nine MIA exposed offspring were considered as one group, regardless of trimester of exposure. MIA exposed offspring did not differ from control animals in weight (t(11)=1.051, *p* = 0.316), but were approximately two months older on average (t(11) = 2.79, p = 0.017) (Table 2). Analysis of [^18^F]FMT signal in the striatum of these nonhuman primates showed that MIA animals had significantly higher [^18^F]FMT index of influx compared to control animals (F_1,11_ = 10.98, *p* = 0.007) (Fig. 1). In spite of the modest sample size, this group difference reflects a large effect size (Cohen’s *d* = 0.998).

## Discussion

The nonhuman primate model is uniquely positioned to explore the confluence of prenatal immune challenge and dopaminergic functioning in a highly translational animal model system. We have previously demonstrated that rhesus monkeys exposed to prenatal immune challenge develop aberrant behaviors after a period of early typical development^17–19^. Here we present initial results from a PET study carried out when these MIA-treated offspring reached 3.5 years of age, which is roughly equivalent to late adolescence in humans. Analysis of [^18^F]FMT signal in the striatum of these nonhuman primates showed that MIA-treated animals had significantly higher [^18^F]FMT index of influx compared to control animals (Fig. 1). These data provide the first evidence of hyperdopaminergia in a nonhuman primate MIA model, which bridges the gap between rodent MIA models and SZ patient populations.

Emerging evidence from rodent MIA models suggests that dopaminergic alterations originating early in fetal development contribute to the emergence of SZ-related behavioral alterations observed as the offspring mature^21^. Rodent offspring born to MIA-treated dams exhibit a multitude of behavioral alterations associated with dopamine system functioning, including potentiation of amphetamine sensitivity, disruption of selective attention, and sensorimotor gating, that can be alleviated with dopamine-blocking compounds^25,41^. Although we are at the early stages of understanding the neurobiological basis of aberrant behavior development in MIA-treated offspring, hyperdopaminergia in the striatum has emerged as one of the more consistent and translationally relevant outcomes. For example, increased stimulated striatal dopamine release has been observed in in vitro slice preparations derived from adult rats born to dams injected with PolyI:C at mid/late gestation (GD 15) compared to controls^23^. Increased dopamine turnover has also been observed in the striatum of adult mice born to dams that received multiple PolyI:C injections from mid to late gestation (GD 12–17)^24^. Furthermore, Meyer and colleagues have demonstrated increases in dopamine-related neurochemical markers, such as tyrosine hydroxylase (TH), in the striatum of adult mice born to dams that received a single PolyI:C injection in early/mid pregnancy (GD 9)^42^. Subsequent studies exploring age- and region-specific dopaminergic alterations have reported an initial decrease in TH immunoreactivity in peripubertal MIA-treated mouse offspring that was followed by an increase in TH immunoreactivity in the ventral (but not dorsal) striatum when the mice reached adulthood^22^. Thus, rodent MIA models have demonstrated abnormalities in dopamine system development that are both region-specific and dependent on the stage of offspring development. Recent evidence suggests that co-administration of vitamin D hormone during pregnancy prevents behavioral deficits^43^ and abnormal dopaminergic phenotypes^44^ in MIA-treated offspring, possibly by exerting neuroprotective effects on the developing dopamine system.

In spite of the mounting evidence of dopamine dysfunction in rodent MIA models, we are unaware of any preclinical studies that have utilized PET imaging to quantify striatal dopamine, as described below in human patient populations. Thus, the present study extends the rodent MIA model findings to a species more closely related to humans and provides a novel model system to further explore the role of aberrant dopaminergic development following prenatal immune challenge. Interestingly, abnormalities in dopamine system development described in MIA-treated nonhuman primates aligns with previous behavioral observations from this same cohort. In an exploratory eye-tracking study, we reported that the first trimester MIA-exposed nonhuman primates failed to attend to salient social features (i.e., the eye region) in a species-typical manner^19^. Given that the mesolimbic dopamine system and the cortical salience network are thought to play a critical role in the detection of behaviorally relevant environmental stimuli^45^, it is plausible that early disturbances in dopaminergic development may alter salience processing abilities. Indeed, aberrant functioning of both the salience network and the mesolimbic dopamine have been observed in numerous neuropsychiatric disorders, including disorders associated with maternal infection, such as SZ^46–49^.

Translationally aligned with our findings, two recent meta-analyses of molecular imaging studies in human patients report elevated striatal dopamine in SZ^50,51^. Previous studies in individuals with SZ suggest that the striatal "hyperdopaminergic" state in the illness is primarily driven by presynaptic (e.g., increased DA synthesis) as opposed to postsynaptic (e.g., increased postsynaptic DA receptor expression) mechanisms^52–59^. Although the precise anatomical locus of dopamine dysfunction within the striatum remains unclear, a recent meta-analysis suggests that dopaminergic dysfunction is greater in dorsal compared to limbic subdivisions of the striatum for patients with SZ^60^. Elevated presynaptic dopamine has also been observed in at-risk subjects^61^ and is correlated with prodromal symptom severity and furthermore may predict transition to psychosis in at-risk patients^62^. The potential clinical utility of presynaptic dopamine as a molecular biomarker for psychosis has also been demonstrated in PET studies comparing treatment responders and non-responders. Specifically, striatal dopamine levels have been shown to predict antipsychotic treatment response in SZ, with striatal dopamine synthesis capacity elevated in treatment responders but not in non-responders^63,64^. Presynaptic dopamine, therefore, may be used to identify patients unlikely to respond to antipsychotic treatment and who therefore may require alternative or additional forms of treatment. Recent studies have also begun to explore the association between childhood adversity (e.g., abuse, multiple families, immigration), elevated striatal dopamine function and increased risk of developing psychosis^65,66^. Although additional research is needed to understand the complex relationship between early life stress and dopamine dysregulation^67^, the MIA model is uniquely positioned to address these questions by incorporating second “hits” such as postnatal stress following prenatal immune challenge^68^.

Taken together with the present findings, these results support striatal presynaptic hyperdopaminergia as a clinically relevant, translational target biomarker for SZ patients and at-risk populations. The present study contributes to mounting evidence implicating dopamine dysregulation as a potential mechanism underlying behavioral abnormalities induced by prenatal immune challenge. The primary limitation of the current study is the small sample size of MIA-treated nonhuman primates. Given that early translational studies tend to have quite high per-subject costs, initial nonhuman primate studies must balance the potential information gained versus ethical and financial factors^69^. Thus, small sample sizes are not uncommon for initial pilot studies in nonhuman primates^70–73^. Moreover, the current study included only male subjects prenatally exposed to MIA, thus we are unable to explore sex differences that have been reported in previous rodent MIA models^41^. Despite these limitations, this initial nonhuman primate PET study extends the findings of dopamine dysregulation in rodent MIA models to a species more closely related to humans, thereby highlighting clinical relevance of the nonhuman primate MIA model for understanding the neurodevelopmental origins of SZ. In future studies, we will take advantage of the unique opportunities provided by this nonhuman primate neurodevelopmental model of psychosis risk to study brain structure, function and chemistry longitudinally, along with more sophisticated cognitive and social behavioral phenotyping, in a new cohort of MIA nonhuman primates. This approach has the potential to identify the relationship between emerging adolescent behavioral disturbances and hyperdopaminergia and to identify changes in brain structure and function and optimiz their measurement in order to provide biomarkers for risk prediction for future longitudinal studies of young people at risk for psychosis.
